# Avocado Oil Nanoemulsion Ameliorates STZ-Induced Diabetic Brain Dysfunction through PI3K/AKT/mTOR-Related Gene Modulation and Attenuation of Apoptosis

**DOI:** 10.1007/s12035-026-06238-4

**Published:** 2026-11-04

**Authors:** Fatma A. Ahmed, Marwa S. Khattab, Faten M. Ibrahim, Aya M. Yassin, Mahmoud Emam, Kawkab A. Ahmed

**Affiliations:** 1 Department of Pathology, Faculty of Veterinary Medicine, Cairo University, Giza, 12211 Egypt; 2 Medicinal and Aromatic Plants Research Department, Pharmaceutical and Drug Industries Research Institute, National Research Centre, Giza, Dokki 12622 Egypt; 3 Department of Biochemistry and Molecular Biology, Faculty of Veterinary Medicine, Cairo University, Giza, 12211 Egypt; 4 Phytochemistry and Plant Systematics Department, National Research Centre, Giza, Dokki 12622 Egypt

**Keywords:** Histopathology, Caspase -3, PI3K, AChE, mTOR, PANX1,AKT1,GSK3β, Diabetes, STZ, Avocado

## Abstract

Diabetes mellitus is associated with progressive neurological complications characterized by hyperglycemia-induced oxidative stress, neuronal apoptosis, and disruption of intracellular survival signaling pathways. This study investigated the neuroprotective effects of avocado cold-pressed oil (ACPO) and its nano-formulation against diabetes-induced cerebral alterations in streptozotocin (STZ)-induced diabetic rats. Seventy adult male Wistar rats were randomly allocated to ten groups (*n = *7/group): normal control (NC), blank nano-formulation vehicle control (BC), metformin control (RC), ACPO control (OC), ACPO nano-formulation control (FC), diabetic positive control (PC), diabetic blank nano-formulation vehicle control (BT), diabetic + metformin (RT; 500 mg/kg), diabetic + ACPO (OT; 500 mg/kg), and diabetic + ACPO nano-formulation (FT; 500 mg/kg). The untreated diabetic group served as the reference for diabetes-associated alterations, whereas the diabetic vehicle-control group was included to account for potential effects of the nano-formulation vehicle. Diabetes was induced by a single intraperitoneal injection of STZ (40 mg/kg). Plasma glucose levels, cerebral gene expression of phosphatidylinositol-3-kinase (PI3K), protein kinase B (AKT1), mechanistic target of rapamycin (mTOR), glycogen synthase kinase-3β (GSK3β), pannexin-1 (PANX1), and acetylcholinesterase (AChE) were evaluated. Histopathological examination and caspase-3 immunohistochemistry were performed to assess neuronal injury and apoptosis. Treatment with ACPO and its nano-formulation significantly reduced plasma glucose levels and modulated key molecular pathways involved in neuronal survival. Both treatments upregulated PI3K, AKT1, and mTOR expression while downregulating GSK3β, PANX1, and AChE compared with untreated diabetic rats. Histopathological examination demonstrated marked preservation of cerebral cortical architecture, accompanied by reduced caspase-3 immunoexpression, indicating attenuation of neuronal apoptosis. The nano-formulation exerted greater effects on the investigated molecular biomarkers than conventional ACPO and produced histopathological and immunohistochemical improvements comparable to those observed with metformin.

**Supplementary Information:**

The online version contains supplementary material available at https://doi.org/10.1007/s12035-026-06238-4.

## Introduction

Diabetes mellitus (DM) is one of the most prevalent metabolic disorders worldwide, affecting approximately 589 million adults and representing a leading cause of morbidity, mortality, and healthcare expenditure [1]. Beyond its well-recognized vascular and metabolic complications, diabetes is increasingly associated with neurological disorders that adversely affect cognitive function and quality of life.

Diabetic encephalopathy (DE) is a progressive neurological complication characterized by cognitive impairment, neuronal degeneration, and structural alterations within the central nervous system. Although its underlying pathogenesis remains incompletely understood and no specific therapy is currently available [2], chronic hyperglycemia is considered the principal driving force behind its development. Persistent hyperglycemia promotes oxidative stress, mitochondrial dysfunction, neuroinflammation, and neuronal apoptosis, ultimately leading to cerebral structural abnormalities, synaptic dysfunction, and cognitive decline [3].

Among the molecular pathways implicated in diabetic encephalopathy, the phosphatidylinositol-3-kinase/protein kinase B/mechanistic target of rapamycin (PI3K/Akt/mTOR) signaling cascade plays a central role in regulating neuronal survival, synaptic plasticity, protein synthesis, autophagy, and apoptosis. Under physiological conditions, activation of PI3K/Akt signaling promotes neuronal survival by stimulating mTOR while simultaneously suppressing pro-apoptotic mediators, including glycogen synthase kinase-3β (GSK3β). However, diabetes-induced oxidative stress impairs PI3K/Akt/mTOR signaling, resulting in activation of GSK3β, enhanced neuronal apoptosis, and progressive neurodegeneration [4]. Consequently, restoration of this pathway has emerged as a promising therapeutic strategy for preventing diabetes-associated neuronal injury.

Pannexin-1 (PANX1), a transmembrane channel protein abundantly expressed in neurons and glial cells, has recently been recognized as an important mediator of neuroinflammation. PANX1 channels facilitate the extracellular release of adenosine triphosphate (ATP), which activates purinergic signaling pathways and promotes inflammatory cytokine production, inflammasome activation, and neuronal injury [5, 6]. Increasing evidence indicates that excessive PANX1 activation contributes to the progression of diabetic encephalopathy by amplifying neuroinflammatory responses and oxidative stress, thereby exacerbating neuronal dysfunction [6, 7]

Acetylcholinesterase (AChE), the enzyme responsible for the hydrolysis of acetylcholine, is essential for regulating cholinergic neurotransmission and maintaining cognitive function [8]. Hyperglycemia-induced oxidative stress and neuroinflammation have been shown to increase AChE expression and activity, leading to reduced acetylcholine availability, impaired synaptic transmission, and cognitive dysfunction [9]. Beyond its classical cholinergic role, AChE has also been implicated in oxidative stress, inflammatory signaling, and apoptosis, suggesting that its dysregulation contributes to the progression of diabetic encephalopathy [10]. Therefore, modulation of AChE represents a potential therapeutic strategy for preserving neuronal integrity and improving cognitive function in diabetes-associated neurodegeneration[11].

Neuronal apoptosis represents another hallmark of diabetic encephalopathy and is considered a major mechanism underlying progressive neuronal loss. Caspase-3 is the principal executioner caspase responsible for the irreversible dismantling of apoptotic cells, whereas the balance between the pro-apoptotic protein Bax and the anti-apoptotic protein Bcl-2 determines neuronal fate. An increased Bax/Bcl-2 ratio promotes mitochondrial dysfunction and activation of caspase-dependent apoptosis, whereas restoration of this balance favors neuronal survival [12]. Collectively, impairment of PI3K/Akt/mTOR signaling together with activation of GSK3β, PANX1, AChE, and apoptotic pathways forms an interconnected molecular network that contributes to the pathogenesis of diabetic encephalopathy.

Given the multifactorial nature of diabetic encephalopathy, naturally derived bioactive compounds have attracted considerable attention because of their antioxidant, anti-inflammatory, and neuroprotective properties. Among these, avocado (*Persea americana*) cold-pressed oil contains abundant monounsaturated fatty acids, tocopherols, carotenoids, phytosterols, and phenolic compounds that exhibit potent antioxidant and anti-inflammatory activities. These bioactive constituents have been reported to improve metabolic homeostasis, attenuate oxidative stress, suppress inflammatory signaling, and protect neuronal tissues from metabolic injury [13]. Consequently, avocado cold-pressed oil (ACPO) may represent a promising natural therapeutic agent capable of modulating multiple pathogenic mechanisms involved in diabetic encephalopathy.

Despite these promising biological properties, the therapeutic efficacy of many plant-derived bioactive compounds is frequently limited by poor aqueous solubility, instability, limited gastrointestinal absorption, and low bioavailability. Nanoformulation technologies have emerged as an effective strategy to overcome these limitations by enhancing the stability, solubility, absorption, controlled release, and tissue distribution of natural compounds while reducing the required dose and minimizing potential toxicity [14]. Nanoencapsulation therefore offers an attractive approach for improving the biological activity and therapeutic efficacy of avocado oil.

Accordingly, the present study aimed to evaluate the neuroprotective effects of avocado cold-pressed oil and its nano-formulation in streptozotocin-induced diabetic rats. Specifically, the study investigated their effects on hyperglycemia, the PI3K/Akt/mTOR/GSK3β signaling pathway, PANX1 and acetylcholinesterase expression, histopathological alterations, and caspase-3 immunohistochemical expression to elucidate the molecular mechanisms underlying their protective effects against diabetes-induced cerebral injury.

## Methods

### Chemicals

Streptozotocin (STZ; sc-200719, ChemCruz®, Santa Cruz Biotechnology, Dallas, TX, USA) was dissolved in freshly prepared 0.1 M citrate buffer (pH 4.5) immediately before intraperitoneal administration. Metformin hydrochloride (500 mg tablets; Minapharm Pharmaceuticals SAE, Cairo, Egypt) was freshly dissolved in distilled water before oral administration. Avocado cold-pressed oil (ACPO) and its nano-formulation were prepared as described below. All solutions were freshly prepared immediately before use to ensure stability and biological activity.

### Extraction of Avocado Cold-pressed Oil (ACPO)

Fresh mature avocado (*Persea americana* Mill.) fruits were thoroughly washed with distilled water, manually peeled, and the seeds were separated from the pulp. The seeds were carefully cleaned to remove adhering pulp residues and processed within one month of collection. Avocado seed oil was extracted by mechanical cold pressing using a hydraulic cold-press unit at the National Research Centre (NRC), Dokki, Giza, Egypt, following the general principles previously described [15, 16].

Mechanical cold pressing is an environmentally friendly (green) extraction technique that employs mechanical compression without the use of organic solvents and operates at relatively low temperatures (generally below 50 °C), thereby minimizing thermal degradation and preserving the natural lipid profile and heat-sensitive bioactive constituents of the oil [15, 17].

The extracted avocado seed oil was filtered through sterile filter paper to remove residual particulates, transferred into sterile amber glass bottles to protect it from photo-oxidation, and stored at 4 °C until use. All experiments were performed using oil obtained from a single extraction batch to minimize batch-to-batch variation and ensure consistency throughout the study [16].

### Phytochemical and Antioxidant Analysis of Avocado Cold-Pressed Oil (ACPO)

The phytochemical composition of avocado cold-pressed seed oil (ACPO) was characterized by gas chromatography–mass spectrometry (GC–MS) through analysis of its unsaponifiable fraction. The antioxidant capacity of ACPO was evaluated by determining its 2,2-diphenyl-1-picrylhydrazyl (DPPH) radical scavenging activity, nitric oxide (NO) radical scavenging activity, and total phenolic content (TPC). GC–MS analysis was performed to identify the major phytochemical constituents of the oil, whereas the antioxidant assays were conducted according to the validated analytical methods previously described in our study [18].

### Nanoemulsion Preparation and Characterization

The avocado cold-pressed seed oil (ACPO) nanoemulsion was prepared using Tween® 80 (Sigma-Aldrich, St. Louis, MO, USA), a non-ionic surfactant, according to the method previously described [19]. The organic phase consisted of ACPO and Tween 80 mixed at a 1:1 (w/w) ratio. This mixture was added dropwise to distilled water (aqueous phase) under continuous vigorous magnetic stirring at 25 °C, resulting in the spontaneous formation of the nanoemulsion.

The prepared nanoemulsion was transferred into sealed amber glass bottles and stored at 4 °C until use. Before each administration, the formulation was gently mixed to ensure a homogeneous dispersion.

The physicochemical properties of the nanoemulsion, including average particle size, polydispersity index (PDI), and zeta potential, were determined using a Nicomp™ 380 ZLS particle size analyzer (Particle Sizing Systems, Santa Barbara, CA, USA) based on dynamic light scattering (DLS). Particle size was measured at a scattering angle of 170°, whereas zeta potential was determined at 18°, according to the method peviously described [20]. The morphology and particle size of the prepared nanoemulsion were further confirmed by transmission electron microscopy (TEM) using a JEM-2100 PLUS microscope (JEOL Ltd., Tokyo, Japan).

### Animals

Seventy male Wistar rats were obtained from the animal facility of the Research Institute of Ophthalmology, Giza, Egypt, weighing 150–200 g at the time of arrival. The animals were acclimatized for two weeks under standard laboratory conditions before initiation of the experiment. The animals were maintained under controlled environmental conditions at a temperature of 25–27 °C, with a 12-h light/12-h dark cycle. They were provided with a commercial pellet diet containing 21% protein (Ibex Company, Egypt) and water *ad libitum*. Animals were monitored daily throughout the experimental period for general health status, behavior, food and water intake, body weight, and blood glucose levels. The study was conducted in accordance with the ARRIVE guidelines and the National Research Council's Guide for the Care and Use of Laboratory Animals and was approved by the Institutional Animal Care and Use Committee (IACUC) [Approval number: Vet CU 25122023878].

### Experimental Design

Following the acclimatization period, animals were balanced by initial body weight to minimize intergroup variability and ensure accurate dose administration. Subsequently, rats were randomly allocated into ten experimental groups (*n = *7/group). Previous studies using STZ-induced diabetic rat models and evaluating brain molecular, biochemical, and histopathological alterations have employed group sizes ranging from 6 to 10 animals per group [21–24] Therefore, a sample size of seven animals per group was selected in the present study to provide sufficient biological variability while adhering to the 3Rs principle of animal research, particularly the reduction of animal use.

Groups 1–5 consisted of non-diabetic animals. The first group served as the normal control (NC) and received normal saline daily via oral gavage. The second group served as the blank control (BC) and received the blank nano-formulation vehicle daily via oral gavage. The third group served as the reference drug control (RC) and received metformin (500 mg/kg) daily via oral gavage. Metformin (500 mg/kg body weight) was included as a reference antidiabetic drug control to provide a standard pharmacological comparator for evaluating the effects of ACPO and its nano-formulation in STZ-induced diabetic rats [25]**.**

The fourth group served as the avocado cold-pressed oil control (OC) and received ACPO (500 mg/kg) daily via oral gavage. The fifth group served as the ACPO nano-formulation control (FC) and received the ACPO nano-formulation at an equivalent ACPO dose of 500 mg/kg daily via oral gavage.

Following two weeks of oral pretreatment with the respective interventions, diabetes was induced in Groups 6–10 by a single intraperitoneal injection of streptozotocin (STZ, 40 mg/kg), after which administration was continued throughout the experimental period. Thus, the experimental design incorporated a pretreatment phase intended to assess preventive potential, followed by continued administration during the diabetic phase to evaluate their subsequent ameliorative effects. The sixth group served as the diabetic positive control (PC). The seventh group served as the diabetic nano-formulation vehicle control (BT) and received the blank nano-formulation vehicle. The eighth group (RT) received metformin (500 mg/kg). The ninth group (OT) received ACPO (500 mg/kg). The tenth group (FT) received the ACPO nano-formulation at an equivalent ACPO dose of 500 mg/kg. The concentration of the nano-formulation was adjusted to provide an amount of active ACPO equivalent to that administered in the free oil treatment. Because treatment was initiated before STZ induction and continued thereafter, the present design does not permit complete separation of preventive effects from therapeutic effects on established diabetes. The experimental duration was selected based on the experimental design previously reported [23], which demonstrated that this period was sufficient to evaluate the therapeutic effects of cold-pressed avocado oil and its nano-formulation in streptozotocin-induced diabetic rats using biochemical, molecular, histopathological, and immunohistochemical assessments. At the end of the 45-day experimental period, all rats were deeply anesthetized with 2% isoflurane before euthanasia by decapitation. Brain tissues were immediately excised, rinsed in ice-cold saline, and processed for biochemical, molecular, histopathological, and immunohistochemical analyses.

### Induction of Experimental Diabetes Mellitus

Experimental diabetes was induced by a single intraperitoneal injection of streptozotocin (STZ; 40 mg/kg body weight), freshly dissolved in ice -cold 0.1 M citrate buffer (pH 4.5), according to the method previously described [26]. Following STZ injection, rats received 5% glucose solution instead of drinking water for 24 h to prevent transient hypoglycemia induced by acute insulin release. Fasting blood glucose levels were measured after overnight fasting using a handheld glucometer (YASEE Bio Medical Inc., Shandong, China). Blood samples were collected from the tail vein at baseline (before STZ administration), and at 72 h and one week after STZ injection. Rats with fasting blood glucose levels ≥ 250 mg/dL at both post-induction time points were considered diabetic and included in the diabetic experimental groups. Following confirmation of diabetes, fasting blood glucose levels were monitored weekly throughout the experimental period according to the recommendations described [27].

### Plasma Glucose Level

At the end of the experimental period, blood samples were collected, and plasma glucose concentration was determined using commercially available enzymatic colorimetric assay kits (Biodiagnostic, Giza, Egypt) based on the glucose oxidase–peroxidase (GOD–POD) method described by Tinder (1969) [28]. Absorbance was measured using a semi-automated clinical chemistry analyzer (Genrui WP21B, Genrui Biotech Inc., Shenzhen, China), and glucose concentrations were expressed as mg/dL according to the manufacturer's instructions.

### Quantitative Real-time PCR (qRT-PCR) and Gene Expression Evaluation

Total RNA was isolated from 100 mg of cerebral cortical brain tissue using a RNeasy Mini Kit (Qiagen, Hilden, Germany; Cat. No. 74104) according to the manufacturer’s instructions. The concentration and purity of the extracted RNA were determined using a NanoDrop ND-1000 spectrophotometer. Complementary DNA (cDNA) was synthesized from purified RNA using the RevertAid First Strand cDNA Synthesis Kit (Thermo Scientific, USA; Cat. No. K1622). Gene expression levels were quantified relative to the reference gene β-actin (ACTB) using iQ SYBR® Green Supermix (Bio-Rad, USA; Cat. No. 1708880) following the manufacturer’s protocol. Primer sequences are provided in Table 1. Quantitative PCR (qPCR) reactions were performed in a total volume of 20 μL containing 1 μL of cDNA and 0.5 μM of each primer. The thermal cycling conditions included an initial denaturation step at 95 °C for 3 min, followed by 40 cycles of denaturation at 95 °C for 15 s, annealing at 60 °C for 30 s, and extension at 72 °C for 30 s. Cycle threshold (Ct) values were obtained using the Bio-Rad iCycler thermal cycler and MyiQ real-time PCR detection system. At the end of each run, melting curve analysis was performed to verify the specificity of the amplified products. All reactions were conducted in triplicate, and a no-template control (NTC) was included. Relative gene expression levels were calculated using the 2^^−ΔΔCt^ method [29].

**Table 1:** Primer sequences for genes analyzed in the study

Gene symbol	Gene description	Product size	Primer Sequence	Accession number/REF	
Housekeeping gene					
ACTB	Beta-actin (β-actin)	275 bp	F: 5'- AGGCTGTGTTGTCCCTGTATG −3' R: 5'- GGCCATCTCTTGCTCGAAGT −3'		> NM_031144.3
PI3K/AKT/mTOR signaling					
PI3K	Phosphatidylinositol- 4,5-bisphosphate 3- kinase	257 bp	F:5ʹ- GTTGGCTTAACCATGCAACTCC-3' R:5ʹ- TAGTGTCTGGACTGGGCTATCT-3'		> NM_001371300.3
Akt1	serine/threonine kinase 1(protein kinase B)	255 bp	F:5ʹ- ACAGCCCTCAAGTACTCATTCC-3' R:5ʹ- CAGCCCGAAGTCCGTTATCTT-3'		> NM_033230.3
mTOR	Mechanistic target of rapamycin kinase	259 bp	F:5' CTTGGAGAACCAGCCCATAAGA-3 R:5'- AGAAAGGACACCAGCCAATGTA-3	> NM_019906.2	
Neuroinflammation biomarkers					
GSK3ß	Glycogen synthase kinase 3 beta	256 bp	F:5'- GGCCACAGGAAGTCAGTTACA-3- R:5'-ACATAGTCCAGCACCAGGTTAAG - 3'	> NM_032080.1	
PANX1	Pannexin -1	248 bp	F:5'-TCTGCTCCGACCTGAAGTTTATC - 3'R:5'-CAGCCGGCAGCTAATGTATTTC −3'	> NM_199397.2	
AChE	Acetylcholinesterase	279 bp	F:5'-CTCGGGTCTATGCCTACATCTTTG −3' R:5'-TCAGGTTCAGGCTCACGTATTG −3'	> NM_172009.3	
Antioxidant markers					
*SOD1*	Superoxide dismutase1	447 bp	F:5′‐GCAGAAGGCAAGCGGTGAAC‐3′ R:5′‐TAGCAGGACAGCAGATGAGT-3′	Ahmed et al., 2024	
*GPX1*	Glutathione peroxidase1	290 bp	F: 5′-CTCTCCGCGGTGGCACAGT-3′ R:5- CCACCACCGGGTCGGACATAC‐3′	> NM_030826.4	

### Histopathology

Brain samples (cerebral cortex) were immersed in 10% neutral-buffered formalin for fixation. The fixed tissues were routinely processed, embedded in paraffin, and sectioned at 3–4 μm thickness. Tissue sections were stained with hematoxylin and eosin (H&E) and examined under a light microscope equipped with an Olympus XC30 digital camera (Olympus, Tokyo, Japan), according to standard histopathological procedures [30].

Histopathological alterations in the cerebral cortex, particularly neuronal changes, were evaluated using a semi-quantitative scoring system ranging from 0 to 4, as described [31]: 0, no detectable injury; 1, slight injury involving < 10% of the examined tissue; 2, mild injury involving 20–30%; 3, moderate injury involving 40–60%; and 4, severe injury involving > 60% of the examined tissue.

To minimize observer bias, histopathological evaluation and scoring were performed by an experienced pathologist who was blinded to the experimental group assignments. The slides were coded before microscopic examination, and the group identities were revealed only after completion of the histopathological assessment.

### Immunohistochemistry

Immunohistochemical detection of caspase-3 was performed on paraffin-embedded cerebral cortex sections obtained from all experimental groups. Sections were incubated with a rabbit polyclonal anti-caspase-3 primary antibody (E-AB-60017; dilution 1:100; Elabscience Biotechnology Inc., USA). The immunostaining procedure was performed using an avidin–biotin–peroxidase complex according to the manufacturer's instructions, as previously described [32]. 3,3′-Diaminobenzidine (DAB) was used as the chromogenic substrate to visualize caspase-3 immunoreactivity, followed by appropriate counterstaining.

Area percent of Caspase-3 immunoreactivity was quantitatively evaluated using ImageJ software (National Institutes of Health, Bethesda, MD, USA). For each animal, three non-overlapping representative fields were assessed at 200 × magnification, and the percentage of caspase-3-positive area was quantified. The mean area percentage obtained from the three fields was calculated for each animal and used for subsequent statistical analysis.

### Statistical Evaluation

Statistical evaluation was conducted using SPSS software, version 26.0 (SPSS Inc., Chicago, IL, USA*).* Gene expression and glucose measurements were analyzed using one-way analysis of variance (ANOVA) after assessment of homogeneity of variances. Duncan’s multiple range test was used for post-hoc comparisons when homogeneity of variances was satisfied, whereas Tamhane’s T2 test was applied when homogeneity of variances was not satisfied. Parametric data were presented as mean ± standard error, and differences were considered statistically significant when *P < *0.05. Nonparametric data as histopathological lesion scores were analyzed using the Kruskal–Wallis test followed by post-Hoc test as Mann–Whitney to detect significance between each two group. A boxplot was used to represent the median and interquartile range.

## Results

### GC–MS Analysis of the Unsaponifiable Fraction of ACPO

**Fig. 1:**
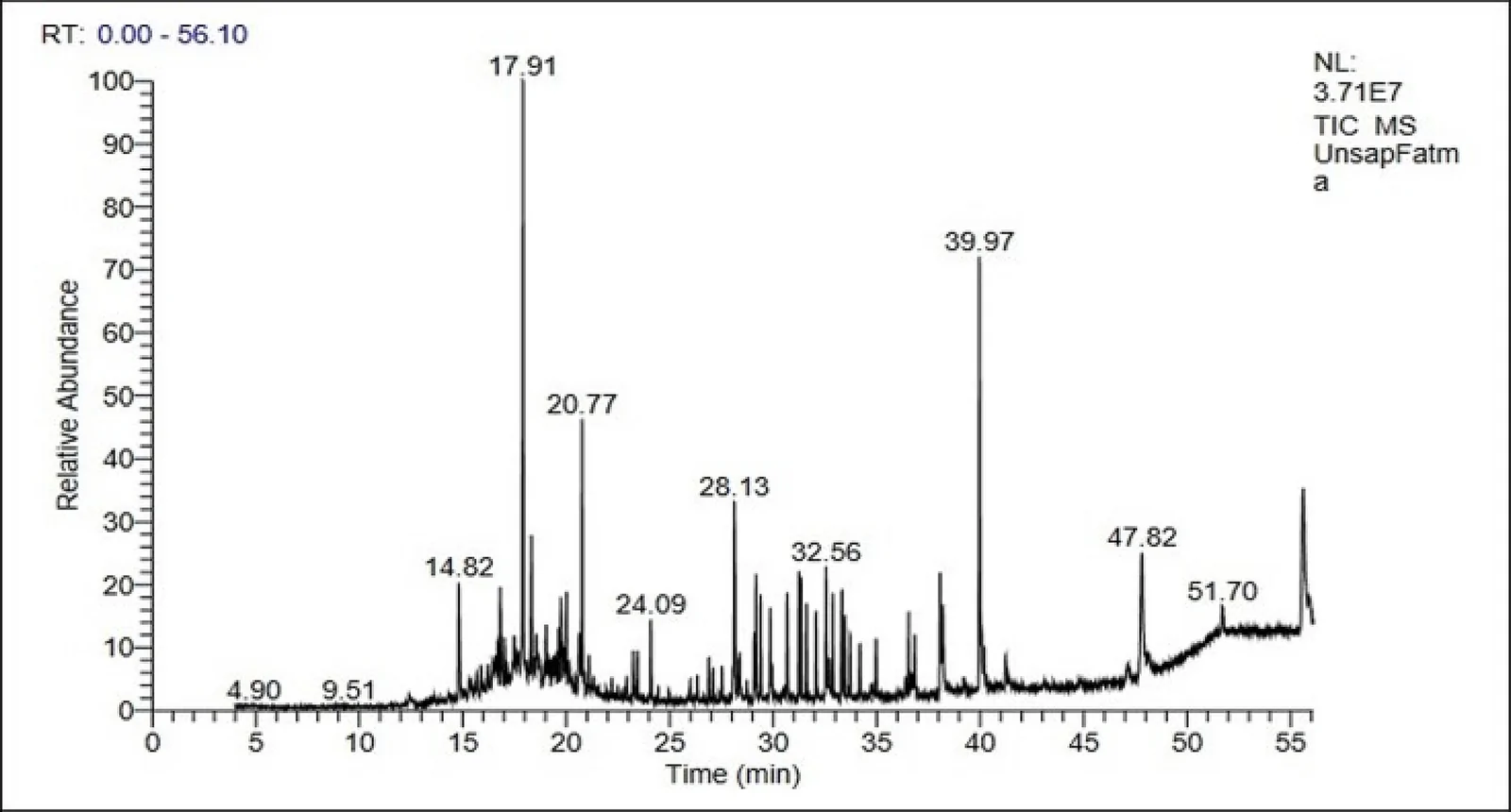
Total ion chromatogram of Unsaponified composition of avocado cold-pressed oil (ACPO) identified by GC–MS analysis

GC–MS analysis of the unsaponifiable fraction of avocado cold-pressed seed oil (ACPO) resulted in the tentative identification of 37 compounds, accounting for 66.03% of the total chromatographic peak area **(**Table 2**; **Fig. 1**).** The identified constituents were predominantly hydrocarbons, including saturated and unsaturated hydrocarbons, together with alkylbenzene derivatives and other minor constituents.

**Table 2:** Chemical compositions of unsaponified matter of avocado fixed oil identified by GC–MS analysis

NO	RT	Conc. %	Identified compounds	Molecular Formula	MW	Chemical Class
1	14.81	3.23	Undecane	C_11_H_24_	15 6	SHC
2	16.67	3.65	3-methyl-Undecane,	C_12_H_26_	17 0	SHC
3	17.02	0.76	2,4-dimethyl- Undecane	C_13_H_28_	18 4	SHC
4	17.49	2.45	Dodecene	C_12_H_24_	16 8	USHC
5	17.91	8.20	Dodecane	C_12_H_26_	17 0	SHC
6	18.32	2.70	6-Methyl-dodecane,	C_13_H_28_	18 4	SHC
7	18.56	2.34	Tridecene		18 2	USHC
8	19.03	1.35	2,4-dimethyl-2- decene	C_12_H_24_	16 8	USHC
9	19.75	1.49	2-Methyl- tetradecane,	C_15_H_32_	21 2	SHC
10	19.94	0.58	2,3- Dimethyldodecane	C_14_H_30_	19 8	SHC
11	20.01	2.00	7-Methyl-tridecane,	C_14_H_30_	19 8	SHC
12	20.63	1.16	2-Methyl naphthalene	C_11_H_10_	14 2	PAHs
13	20.77	4.15	Tridecane	C_13_H_28_	18 4	SHC
14	23.24	0.60	Tetradecene	C_14_H_30_	19 6	USHC
15	23.44	0.66	Tetradecane	C_14_H_30_	19 8	SHC
16	24.09	1.08	Caryophyllene	C_15_H_24_	20 4	bicyclical sesquiterpene US
17	26.89	1.70	5-Phenyl decane Benzene,(1- butylhexyl)-	C_16_H_26_	21 8	Alkyl Benzene derivative
18	28.38	1.33	2-Phenyl decane Benzene, (1-methylnonyl)-	C_16_H_26_	21 8	Alkyl Benzene derivative
19	29.09	0.55	6-Phenyl undecane Benzene, (1 pentylhexyl)-	C_17_H_28_	23 2	Alkyl Benzene derivative
20	29.17	1.18	5-Phenyl undecane Benzene,(1- butylheptyl)-	C_17_H_28_	23 2	Alkyl Benzene derivative
21	29.4	1.02	4-Phenyl undecane Benzene,(1- propyloctyl)-	C_17_H_28_	23 2	Alkyl Benzene derivative
22	29.86	1.57	3-Phenyl undecane Benzene, (1- ethylnonyl)-	C_17_H_28_	23 2	Alkyl Benzene derivative
23	30.68	1.55	2-Phenyl undecane	C_17_H_28_	23 2	Cyclic OHC
24	31.25	1.09	6-Phenyl dodecane Benzene, (1- pentylheptyl)-	C_18_H_30_	24 6	AlkylBenzene derivative
25	31.35	1.09	5-Phenyl dodecane Benzene, (1-butyloctyl)-	C_18_H_30_	24 6	AlkylBenzene derivative
26	31.61	0.93	4-Phenyl dodecane Benzene, (1- propylnonyl)-	C_18_H_30_	24 6	Alkyl Benzene derivative
27	32.07	0.90	3-Phenyl Dodecane Benzene, (1- ethyldecyl)-	C_18_H_30_	24 6	Alkyl Benzene derivative
28	32.56	2.03	3-Eicosene	C_20_H_40_	28 0	USHC
29	32.88	1.32	2-Phenyl Dodecane Benzene, (1- methylundecyl)-	C_18_H_30_	24 6	Alkyl Benzene derivative
30	33.32	1.43	6-Phenyl Tridecane Benzene, (1- pentyloctyl)-	C_19_H_32_	26 0	Alkyl Benzene derivative
31	33.46	0.78	5-Phenyl Tridecane Benzene, (1- butylnonyl)-	C_19_H_32_	26 0	Alkyl Benzene derivative
32	33.72	0.62	4-Phenyl Tridecane Benzene, (1- propyldecyl)-	C_19_H_32_	26 0	Alkyl Benzene derivative
33	34.19	0.68	3-Phenyl Tridecane Benzene, (1- ethylundecyl)-	C_19_H_32_	26 0	Alkyl Benzene derivative
34	34.97	1.14	2-Phenyl Tridecane	C_19_H_32_	26 0	Alkyl Benzene derivative
35	36.55	1.28	1-Docosene	C_22_H_44_	30 8	USHC
36	39.97	4.46	5α,17α-Pregnan-20-one	C_21_H_34_O	30 2	Steroid (C21 pregnane)
37	40.19	2.98	17-Pentatriacontene	C35H70		USHC
Total identified compounds		**66.0 3**				

*RT* retention time, Total identified compound area is 66.03%.: concentration, *SHC* saturated hydrocarbon, *USHC* unsaturated hydrocarbon, *OHC* oxygenated hydrocarbon, *PAHs* polycyclic aromatic hydrocarbons,* MW* Molecular weight

Among the identified compounds, dodecane (8.20%) was the most abundant constituent, followed by tridecane (4.15%). Several alkylbenzene derivatives were also detected, generally at lower individual relative abundances. 5α,17α-Pregnan-20-one represented 4.46% of the total peak area according to the GC–MS library identification. Other identified constituents included caryophyllene (1.08%), 3-eicosene (2.03%), and 17-pentatriacontene (2.98%).

The complete GC–MS profile, including retention time, relative concentration, molecular formula, molecular ion, and chemical classification, is presented in Table 2.

### Avocado Oil Nanoformulation (Nano Emulsion)

**Fig. 2:**
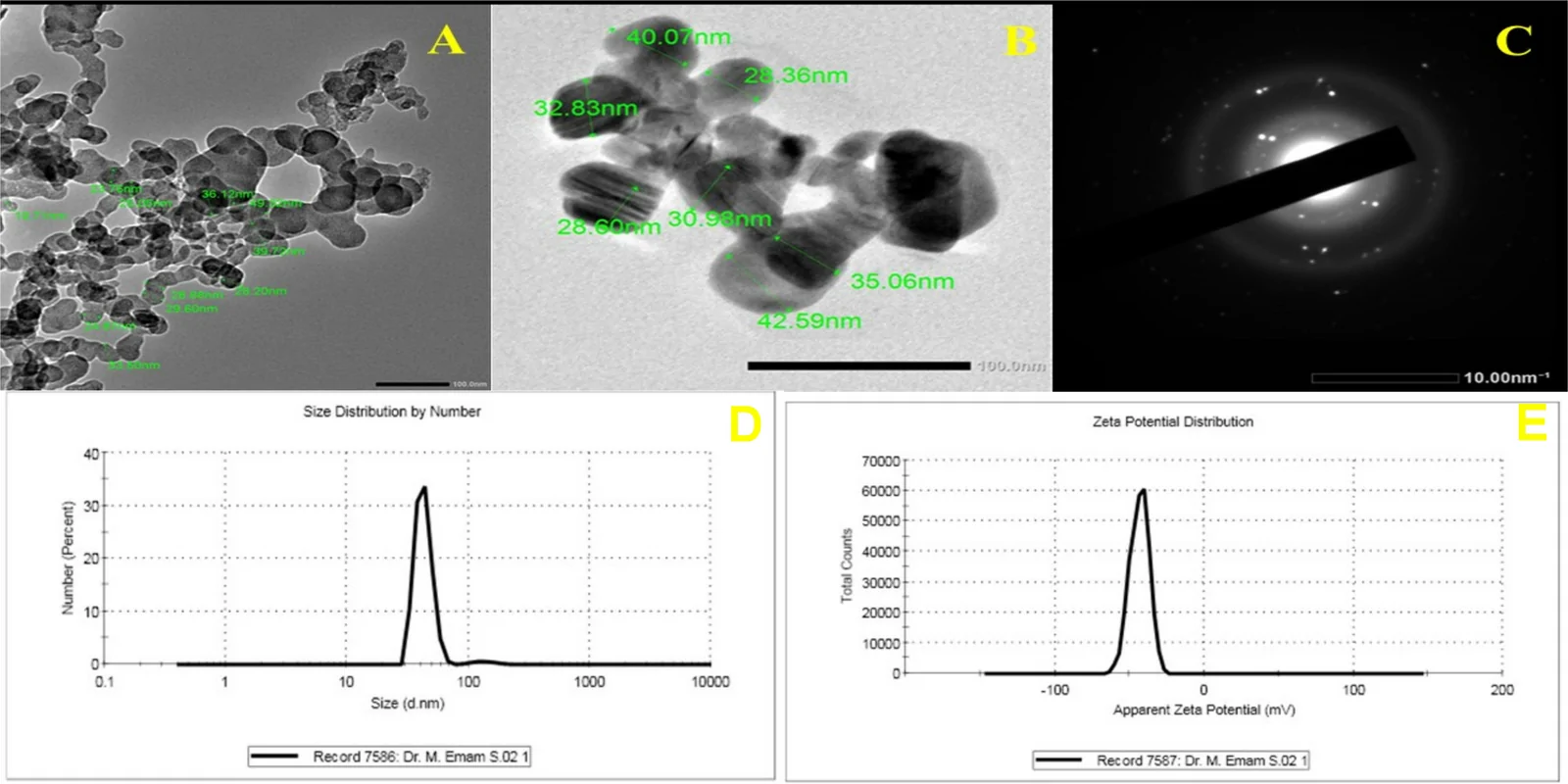
Characterization of the ACPO nanoemulsion using transmission electron microscopy (TEM) (**A** and **B**), selected-area electron diffraction (SAED) (**C**), dynamic light scattering size distribution by number (**D**), and zeta potential distribution (**E**)

The particle size distribution and zeta potential of the formulated avocado oil nanoemulsion were characterized using dynamic light scattering (DLS) (Fig. 2D and E). The number-based DLS analysis showed a major population of 42.89 ± 6.85 nm, representing 97.8% of the distribution by number, together with a minor population of 138.2 ± 30.27 nm, representing 2.2%. The zeta potential was −43.3 mV. The major population identified in the number-based DLS distribution was within the nanoscale range and was broadly consistent with the particle dimensions observed by TEM (Fig. 2A-C).

### Plasma Glucose Concentration

**Fig. 3:**
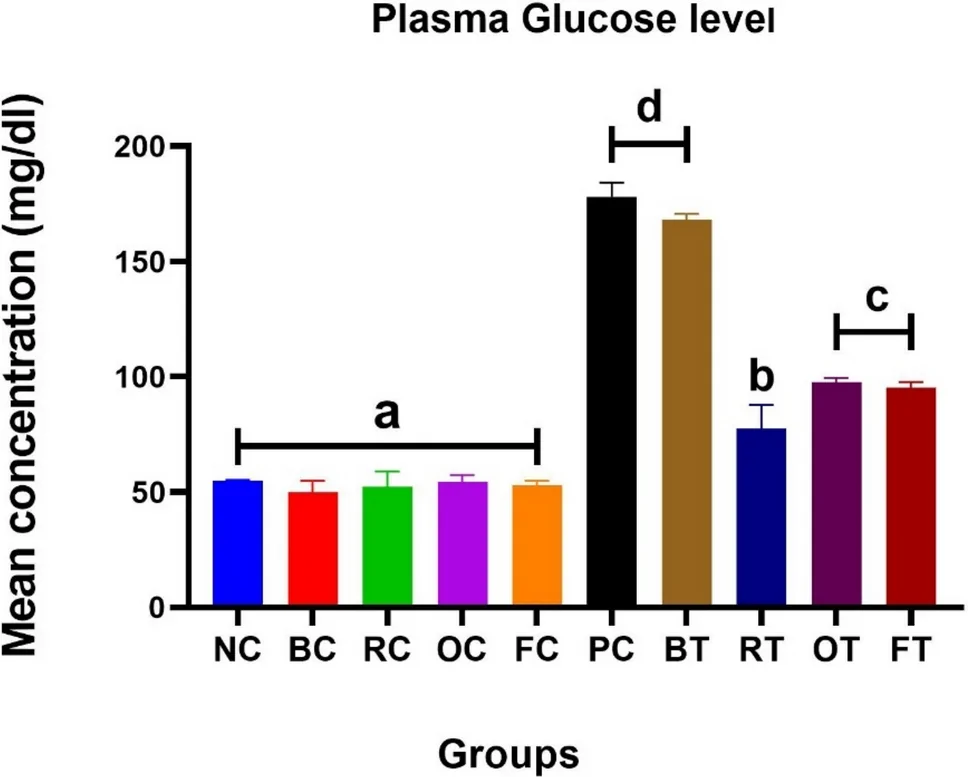
Effect of different treatment models on the plasma glucose level (mg/dl). Data are presented as mean ± standard error (*n = *7). Dissimilar lowercase letters refer to a substantial difference at *P ≤ 0.05* compared to the normal control*.* NC: normal control, BC: blank control, RC: reference drug control, OC: oil control, FC: formula control, PC: positive control, BT: blank treated, RT: reference drug-treated, OT: oil treated, FT: formula treated

The plasma glucose level was remarkably decreased in groups treated with metformin (RT), (OT), and its nano formulation (FT) relative to the positive control group (PC) (Fig. 3).

### Gene Expression Analysis

#### Cerebral PI3K/AKT/mTOR Signaling

**Fig. 4:**
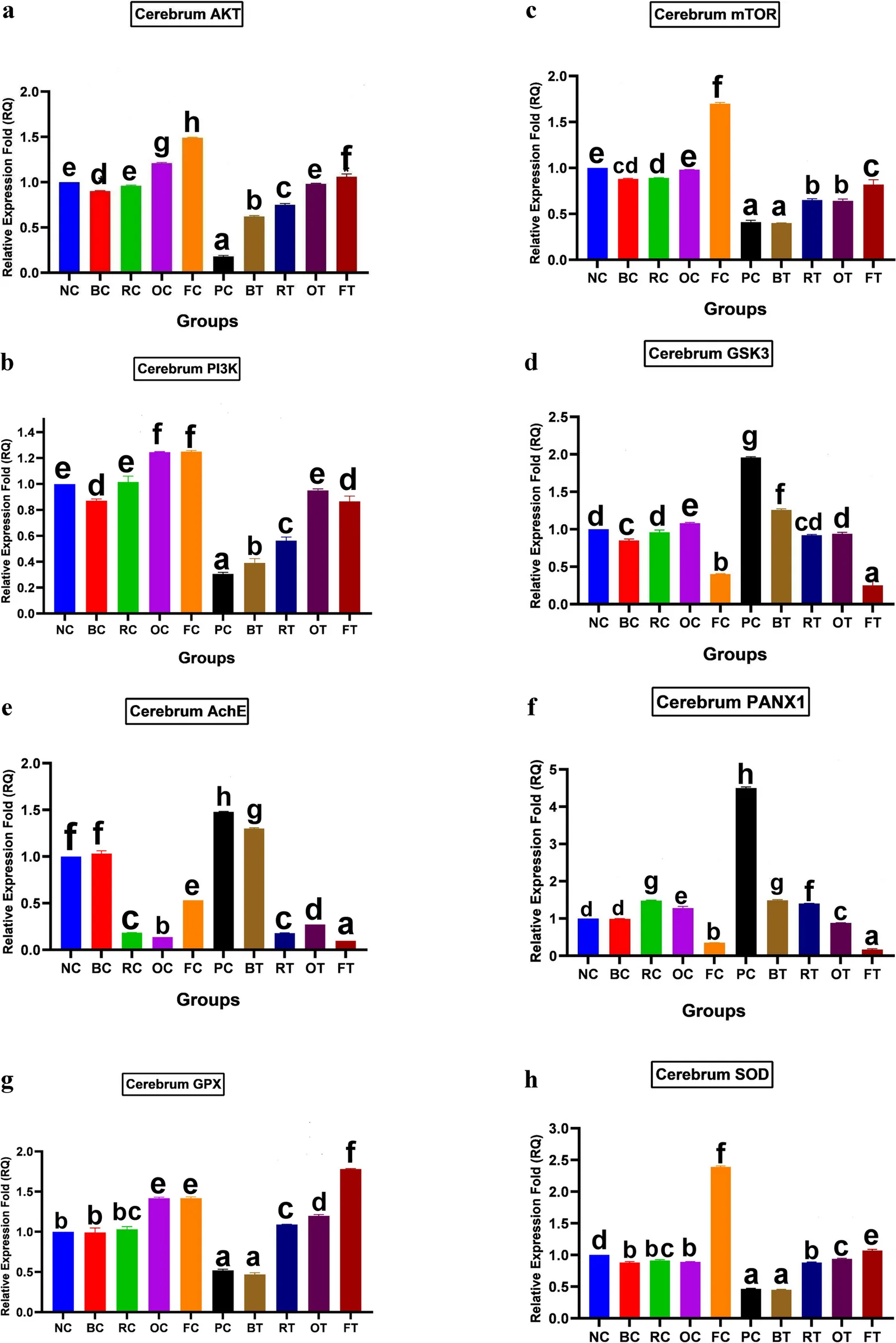
Effect of different treatments on the relative expression level of cerebral: (**a**) Akt, (**b**) PI3K, (**c**) mTOR, (**d**) GSK3ß, (**e**) acetylcholine esterase (AChE) , (**f**) PANX1, (**g**) SOD1 (superoxide dismutase1), (**h**) GPX1 (glutathione peroxidase1) genes in STZ- induced diabetes rat model. Data are presented as mean ± standard error (*n = *7). Different lowercase letters indicate statistically significant differences among groups at *P ≤ *0.05. NC: normal control; BC: blank control; RC: reference drug control; OC: oil control; FC: formula control; PC: positive control; BT: blank treated; RT: reference drug-treated; OT: oil-treated; FT: formula-treated

PI3K gene expression was significantly downregulated (*P < *0.05) in the PC and BT groups compared with all other groups. Treatment with metformin, ACPO, and the ACPO nano-formulation significantly upregulated PI3K gene expression compared with the diabetic control groups (*P < *0.05). Among the diabetic treatment groups, the ACPO-treated group (OT) exhibited the greatest upregulation of PI3K gene expression, showing significantly higher expression than both the ACPO nano-formulation (FT) and metformin-treated (RT) groups (*P < *0.05). Furthermore, the FT group also exhibited significantly higher PI3K gene expression than the RT group (*P < *0.05) **(**Fig. 4b).

AKT1 gene expression was likewise significantly downregulated (*P < *0.05) in the PC and BT groups compared with all other groups. Treatment with metformin, ACPO, and the ACPO nano-formulation significantly upregulated AKT1 gene expression compared with the diabetic control groups (*P < *0.05). The ACPO nano-formulation (FT) produced the greatest upregulation of AKT1 gene expression among the diabetic treatment groups, exhibiting significantly higher expression than both the ACPO-treated (OT) and metformin-treated (RT) groups (*P < *0.05). Moreover, the OT group also exhibited significantly higher AKT1 gene expression than the RT group (*P < *0.05) (Fig. 4a).

Similarly, mTOR gene expression was significantly downregulated (*P < *0.05) in the PC and BT groups compared with all other groups. Treatment with metformin, ACPO, and the ACPO nano-formulation significantly upregulated mTOR gene expression compared with the diabetic control groups (*P < *0.05). The ACPO nano-formulation (FT) demonstrated the highest mTOR gene expression among the diabetic treatment groups, exhibiting significantly higher expression than both the ACPO-treated (OT) and metformin-treated (RT) groups (*P < *0.05). No significant difference was observed between the OT and RT groups (Fig. 4c).

#### Neuroinflammatory Biomarkers

GSK3β gene expression was significantly upregulated (*P < *0.05) in the PC and BT groups compared with all other groups. Treatment with metformin, ACPO, and the ACPO nano-formulation significantly decreased GSK-3β gene expression compared with the diabetic control groups (*P < *0.05). Among the diabetic treatment groups, the ACPO nano-formulation (FT) exhibited the greatest downregulation of GSK3β gene expression, showing significantly lower expression than both the ACPO-treated (OT) and metformin-treated (RT) groups (*P < *0.05). No significant difference was observed between the OT and RT groups (Fig. 4d).

Similarly, AChE expression showed a markedly different pattern (*P < *0.05). Expression was substantially increased in PC and BT compared with the non-diabetic and treated groups. Metformin, ACPO, and the ACPO nano-formulation significantly reduced AChE expression relative to the diabetic control groups. Among the diabetic treatment groups, FT exhibited the lowest AChE expression, followed by RT and OT. OT showed significantly higher AChE expression than RT (*P < *0.05) (Fig. 4e).

Likewise, PANX1 gene expression was significantly upregulated (*P < *0.05) in the PC and BT groups compared with all other groups. Treatment with metformin, ACPO, and the ACPO nano-formulation significantly downregulated PANX1 gene expression compared with the diabetic control groups (*P < *0.05). The ACPO nano-formulation (FT) produced the greatest downregulation of PANX1 gene expression among the diabetic treatment groups, exhibiting significantly lower expression than both the ACPO-treated (OT) and metformin-treated (RT) groups (*P < *0.05). Furthermore, the OT group exhibited significantly lower PANX1 gene expression than the RT group (*P < *0.05) (Fig. 4f).

#### Cerebral Antioxidant Markers

GPx1 gene expression was significantly downregulated (*P < *0.05) in the PC and BT groups compared with all other groups. Treatment with metformin, ACPO, and the ACPO nano-formulation significantly upregulated GPx1 gene expression compared with the diabetic control groups (*P < *0.05). Among the diabetic treatment groups, the ACPO nano-formulation (FT) exhibited the greatest upregulation of GPx1 gene expression, showing significantly higher expression than both the ACPO-treated (OT) and metformin-treated (RT) groups (*P < *0.05). Furthermore, the OT group also exhibited significantly higher GPx1 gene expression than the RT group (*P < *0.05) (Fig. 4g).

Similarly, SOD1 gene expression was significantly downregulated (*P < *0.05) in the PC and BT groups compared with all other groups. Treatment with metformin, ACPO, and the ACPO nano-formulation significantly upregulated SOD1 gene expression compared with the diabetic control groups (*P < *0.05). The ACPO nano-formulation (FT) demonstrated the greatest upregulation of SOD1 gene expression among the diabetic treatment groups, exhibiting significantly higher expression than both the ACPO-treated (OT) and metformin-treated (RT) groups (*P < *0.05). Furthermore, the OT group also exhibited significantly higher SOD1 gene expression than the RT group (*P < *0.05) (Fig. 4h).

### Histopathology

**Fig. 5:**
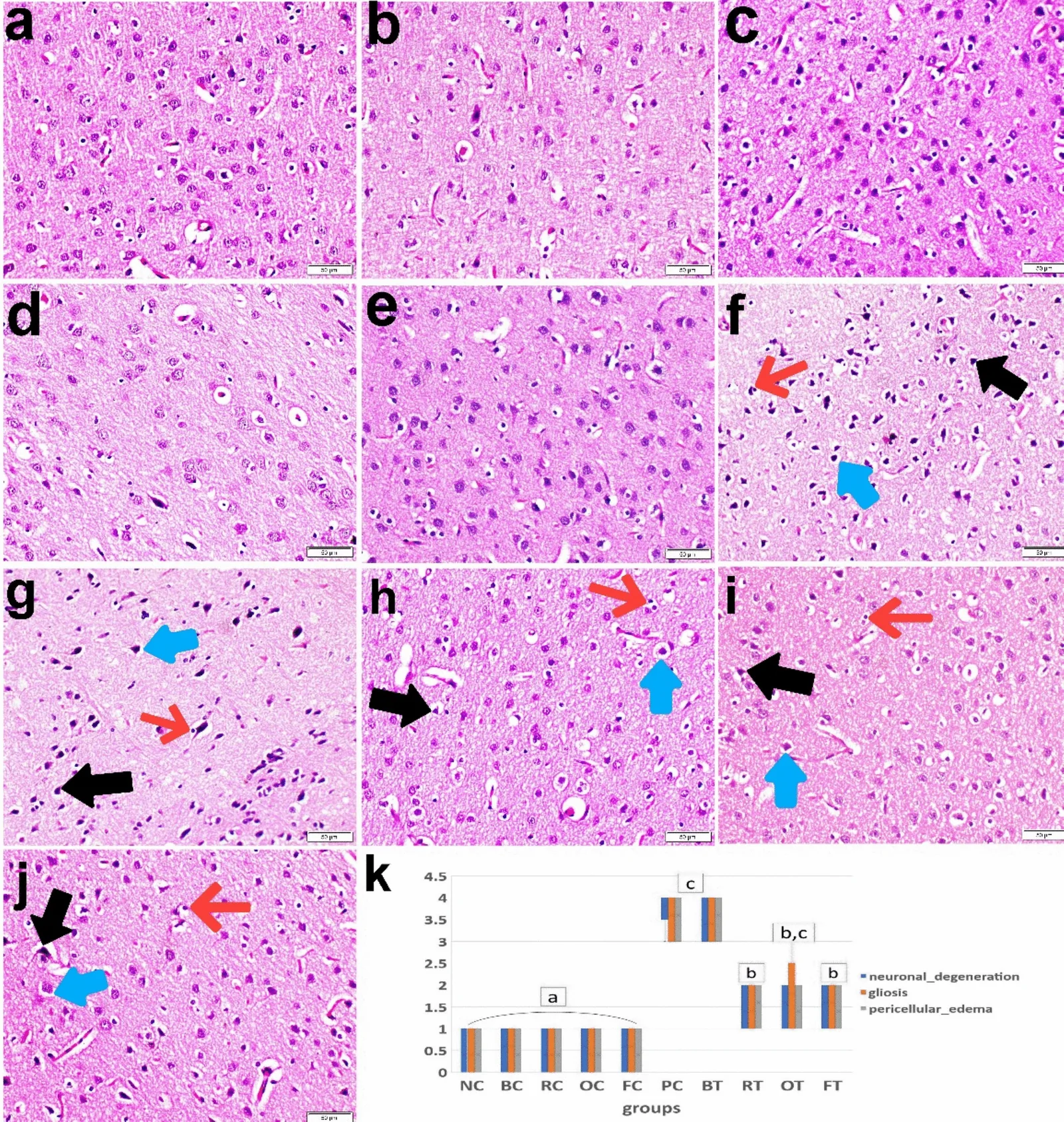
Histopathology of cerebral cortex examined at 200X. (**a**) Normal (NC), (**b**) blank (BC), (**c**) reference drug (RC), (**d**) avocado cold-pressed oil (OC), and (**e**) nano-formulation of avocado cold-pressed oil (FC) control groups showed normal histological structure. (**f**) Positive control (PC), and (**g**) blank treated (BT) showed several necrotic neurons and pericellular edema. (**h**) Reference drug (RT), (**i**) avocado cold-pressed oil (OT), and (**j**) nano- formulation of avocado cold-pressed oil (FT) treated groups showed a low number of necrotic neurons with pericellular edema. (**k**) Boxplot of histopathological score in which the middle thick lines represented the median and the thin lines represented the minimum and maximum values. Black arrows referred to necrotic neurons. Blue arrows referred to pericellular edema. Red arrows referred to gliosis. Data are presented as mean ± standard error (*n = *7). Dissimilar lowercase letters indicate a substantial difference at *P ≤ *0.05 compared to the normal control (scale bar 50 µm)

The control groups exhibited a normal histological architecture of the cerebral cortex (Fig. 5a–e). In contrast, microscopic examination of the cerebral cortex in the diabetic groups, namely, the positive control (PC) and blank-treated (BT) groups, revealed neuronal degeneration characterized by pyknotic nuclei, pericellular edema, and vacuolation of the neuropil (Fig. 5f, g). Additional pathological alterations included congestion of neuronal capillaries, neuronophagia, and gliosis. The RT and OT groups showed mild neuronal degeneration, along with edema and vacuolation in the neuropil. Notably, the RT, OT, and FT groups demonstrated marked histological improvement in the cerebral cortex, with only mild degeneration observed in a few neurons (Fig. 5h–j).

### Immunohistochemistry

**Fig. 6:**
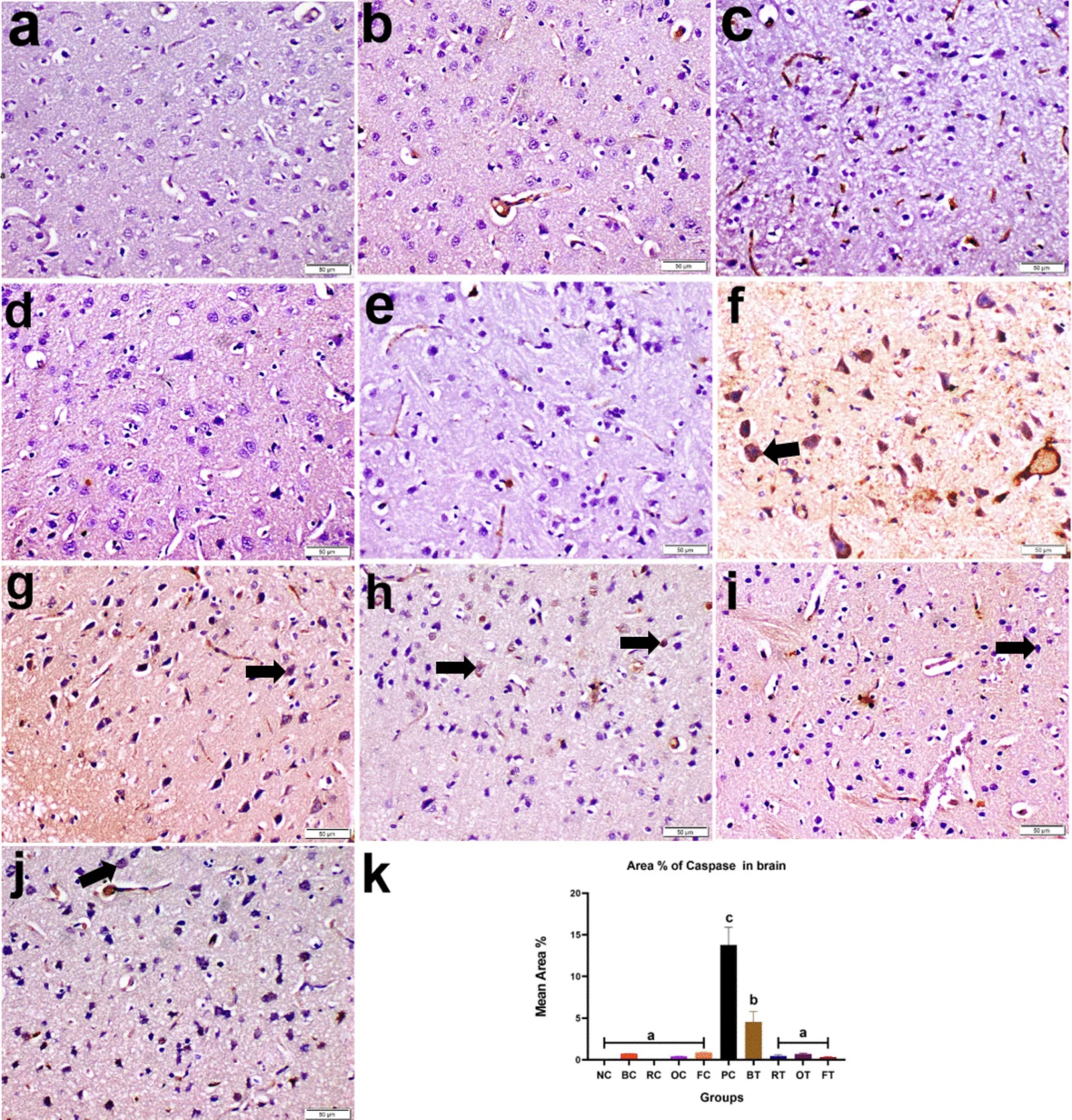
Immune expression of caspase-3 in the brain examined at 200X with determination of the positive area percentage. (**a**) Normal (NC), (**b**) blank (BC), (**c**) reference drug (RC), (**d**) avocado cold-pressed oil (OC), and (**e**) nano-formulation of avocado cold-pressed oil control (FC) groups exhibited no reaction. (**f**) Positive control (PC) and (**g**) blank- treated (BT) groups exhibited a strong reaction. (**h**) Reference drug (RT), (**i**) avocado cold-pressed oil (OT), and (**j**) nano-formulation of avocado cold- pressed oil (FT) treated groups showed a mild reaction. Black arrows refer to the immune reaction represented by the brown color. (**k**) Mean area percentage of caspase-3-positive expression in the brain. Data are presented as mean ± standard error (*n = *7). Dissimilar lowercase letters indicate a substantial difference at *P ≤ *0.05 compared to the normal control (scale bar 50 µm)

The protein expression of caspase-3 in the cerebral tissue of STZ-diabetic rats is illustrated in Fig. 6. In non-diabetic rats, caspase-3 was weakly expressed in neurons (Fig. 6a–e). In contrast, strong caspase-3 expression levels were detected in the cortical neurons of the positive control and blank-treated groups, appearing as dark brown granules within neuronal cells (Fig. 6f, g). The treated groups (RT, OT, and FT) exhibited moderate caspase-3 expression (Fig. 6h–j).

Quantitative image analysis demonstrated a significant increase in caspase-3-positive area percent in the PC group compared with all other experimental groups (*P < *0.05). The BT group also showed significantly higher caspase-3-positive area percent than the control and treatment groups. In contrast, no significant differences were observed among the NC, BC, RC, OC, FC, RT, OT, and FT groups (*P > *0.05), indicating that treatment with metformin, ACPO, or the ACPO nano-formulation reduced caspase-3 immunoreactivity toward levels comparable to those observed in the non-diabetic groups (Fig. 6k).

## Discussion

GC/MS analysis of the unsaponifiable matter of avocado fixed oil revealed the identification of 37 compounds, contributing for 66.03% of the total peak area. Most of these compounds belonged to straight-chain hydrocarbons and phenyl hydrocarbons. The major constituents were dodecane (8.20%), tridecane (4.15%), and Steroid (C21 pregnane) (4.46%), represented by 5α,17α-pregnan-20, and this is in agreement with [33]. The nanoemulsion exhibited droplet sizes in the range of 20–200 nm [34]. The nanoemulsion exhibits a small droplet size when the hydrophile–lipophile balance (HLB) value of the surfactant couple matches the HLB value of the oil [35]. Moreover, the surface charge of nanoemulsion droplets, reflected by the zeta potential, can influence colloidal stability by affecting electrostatic interactions between dispersed droplets [36]. Zeta potential values exceeding approximately ± 30 mV are generally considered favorable for electrostatic stabilization of colloidal systems [37]. In the present study, the nanoemulsion exhibited an average zeta potential of − 43.3 mV, suggesting that electrostatic repulsion may contribute to maintaining dispersion stability and reducing the tendency of droplets to aggregate. However, the relatively high polydispersity index (PDI = 0.6) indicates a broad droplet size distribution, and therefore the zeta potential value alone should not be interpreted as evidence of high or long-term physical stability. Accordingly, the DLS findings support the presence of a nanoscale dispersed system with a substantial negative surface charge, while dedicated stability studies would be required to confirm its stability over time [37].

The neurological complications of diabetes mellitus are closely associated with impaired neuronal survival signaling and cognitive dysfunction. Among the major intracellular pathways involved, the PI3K/Akt/mTOR signaling cascade plays a central role in regulating neuronal survival, protein synthesis, synaptic plasticity, and cellular metabolism. Under physiological conditions, insulin activates the PI3K/Akt pathway, promoting neuronal viability and normal cognitive function [38]. In diabetes mellitus, insulin deficiency and insulin resistance impair activation of this pathway, resulting in reduced PI3K, Akt, and mTOR gene expression, ultimately contributing to neuronal apoptosis and cognitive decline [39]. In the present study, diabetic rats exhibited significant downregulation of PI3K, Akt, and mTOR gene expression, whereas administration of ACPO and its nano-formulation significantly restored the expression of these pathway-related genes. Compared with metformin, the ACPO nano-formulation produced significantly higher Akt and mTOR gene expression, whereas free ACPO induced the highest PI3K gene expression. These findings suggest that nanoencapsulation enhanced the biological efficiency of ACPO by promoting a more pronouced modulation of PI3K/Akt/mTOR pathway-related gene expression. The improved aqueous dispersibility, stability, and bioavailability associated with nanoencapsulation may facilitate more efficient intracellular delivery of ACPO bioactive phytoconstituents, potentially supporting prosurvival molecular responses and contributing to the attenuation of diabetes-associated neuronal dysfunction [7, 40].

Comparison with metformin provides further insight into the nature of these effects. Although metformin produced a greater reduction in blood glucose, the ACPO nano-formulation produced stronger modulation of several cerebral molecular endpoints, whereas responses were comparable between treatments for other parameters. This divergence suggests that the cerebral effects of ACPO nano-formulation were not solely dependent on glycemic control and may additionally involve antioxidant and cell-survival mechanisms associated with its phytochemical composition. Metformin itself has recognized neuroprotective properties and can modulate PI3K/AKT-related signaling [41] therefore, the present findings should not be interpreted as demonstrating universal superiority of ACPO nano-formulation over metformin. Rather, they indicate that the nano-formulation produced a distinct and multifaceted cerebral response under the experimental conditions of this study.

Pannexin-1 (PANX1) channels are widely expressed in neurons and glial cells and play an important role in neuroinflammation by promoting ATP release, inflammatory cell recruitment, and pro-inflammatory mediator production following neuronal injury [42]. Consistent with these observations, diabetic rats in the present study exhibited significant upregulation of PANX1 gene expression, whereas treatment with ACPO and its nano-formulation significantly reduced its expression. Notably, the ACPO nano-formulation produced significantly lower PANX1 gene expression than metformin, suggesting more effective attenuation of diabetes-induced neuroinflammatory signaling. This enhanced effect may be related to the physicochemical characteristics of the nano-formulation, which may improve the dispersion and biological availability of ACPO bioactive phytoconstituents and facilitate their interaction with cellular targets involved in inflammatory pathways [43]. However, direct brain delivery, biodistribution, and intracellular concentrations of these constituents were not assessed in the present study, and therefore enhanced brain delivery cannot be confirmed.

GSK3β is a key regulator of insulin signaling, neuronal survival, and apoptosis. Increased GSK-3β activity contributes to insulin resistance through inhibitory phosphorylation of insulin receptor substrate-1 (IRS-1), thereby aggravating neuronal dysfunction under diabetic conditions [44]. In agreement with previous reports, diabetic rats showed significant upregulation of GSK3β gene expression, whereas treatment with ACPO and its nano-formulation significantly reduced its expression. Importantly, the ACPO nano-formulation resulted in significantly lower GSK3β gene expression than metformin, indicating more effective restoration of insulin-related signaling and neuronal survival. This effect may be associated with the enhanced physicochemical and dispersibility characteristics of the nano-formulation, which may improve the biological availability and cellular interaction of MUFA- and PUFA-rich phytoconstituents of avocado oil, thereby potentially facilitating modulation of insulin-related signaling and suppression of GSK-3β gene expression [45, 46]. Acetylcholinesterase (AChE) plays a central role in terminating cholinergic neurotransmission through hydrolysis of acetylcholine. Increased AChE expression has been associated with diabetes-induced neuronal dysfunction and cognitive impairment [47]. In the present study, both ACPO and its nano-formulation significantly downregulated AChE gene expression compared with diabetic rats. Moreover, the ACPO nano-formulation produced significantly lower AChE gene expression than metformin, suggesting greater preservation of cholinergic function. The enhanced molecular efficacy of the nano-formulation may be related to improved delivery and bioavailability of ACPO bioactive constituents following nanoencapsulation, leading to more effective attenuation of oxidative stress and neuroinflammatory processes that contribute to cholinergic dysfunction [48, 49]. The ACPO nano-formulation produced significantly higher GPx and SOD gene expression than metformin, indicating greater modulation of endogenous antioxidant defense-related gene expression. Upregulation of these antioxidant genes may reduce oxidative stress by limiting reactive oxygen species accumulation and preserving cellular redox homeostasis, thereby protecting neurons from diabetes-induced oxidative damage. The enhanced antioxidant response observed with the nano-formulation may be attributed to the improved bioavailability and intracellular delivery of the bioactive phytoconstituents of avocado oil following nanoencapsulation. Avocado oil is naturally rich in antioxidant compounds, including carotenoids, tocopherols, phenolic compounds, and monounsaturated fatty acids, which have been reported to enhance antioxidant defenses and protect against oxidative stress [50].

Although direct blood–brain barrier (BBB) penetration was not evaluated in the present study, the observed neuroprotective effects, including modulation of multiple molecular pathways, reduced caspase-3 immunoexpression, and improved histopathological findings, suggest that the ACPO nano-formulation exerted biological effects within the brain. Under diabetic conditions, BBB integrity may be compromised as a consequence of oxidative stress, neuroinflammation, and endothelial dysfunction, potentially facilitating nanoparticle interaction with cerebral tissue [51]. Moreover, the nano-formulation exhibited a small mean particle size (90.5 nm), as determined by DLS and confirmed by TEM analysis, which may enhance brain delivery through proposed mechanisms such as passive diffusion across compromised BBB regions, adsorptive-mediated transcytosis, or receptor-mediated endocytosis [52]. Nevertheless, these mechanisms remain hypothetical and were not experimentally investigated in the present study. Therefore, further pharmacokinetic studies, biodistribution analyses, and dedicated BBB permeability experiments are required to confirm brain penetration and elucidate the exact transport mechanism of the ACPO nano-formulation.

Histopathological examination demonstrated that both ACPO and its nano-formulation markedly ameliorated diabetes-induced cerebral lesions, including neuronal degeneration and gliosis, compared with the diabetic group. These findings are consistent with previous reports demonstrating that the antioxidant and anti-inflammatory phytoconstituents of avocado oil, particularly oleic acid and other monounsaturated fatty acids, can attenuate oxidative stress and neuroinflammation, thereby preserving neuronal integrity [48]. In addition, avocado oil contains lutein, which has been reported to modulate mitochondrial function through regulation of transcription factors such as NRF-1 and PGC-1α, contributing to neuronal protection [53]. Although the ACPO nano-formulation produced greater modulation of several molecular markers than metformin, both treatments resulted in comparable histopathological improvement, suggesting that enhanced molecular regulation by the nano-formulation ultimately translated into a similar degree of structural neuroprotection under the present experimental conditions.

The immunohistochemical findings further supported the histopathological observations. Caspase-3 is a key executioner caspase that mediates the final stages of apoptosis and is markedly activated under diabetic conditions [12, 54]. In the present study, diabetic rats exhibited significantly increased caspase-3 immunoexpression, whereas treatment with ACPO and its nano-formulation markedly reduced its expression, indicating attenuation of neuronal apoptosis. These anti-apoptotic effects are likely related to the rich antioxidant phytoconstituents of avocado oil, including flavonoids, tocopherols, phenolic compounds, carotenoids, and monounsaturated fatty acids, which have been shown to protect neurons against oxidative stress-induced apoptosis [55]. Notably, the ACPO nano-formulation and metformin produced comparable reductions in caspase-3 immunoexpression, indicating that both treatments achieved a similar anti-apoptotic effect despite the superior molecular modulation observed with the nano-formulation.

In addition to its neuroprotective effects, the ACPO nano-formulation did not produce evidence of treatment-related hepatic or renal toxicity under the present experimental conditions. Serum biochemical analyses (Supplementary Figure S1) together with histopathological examination of the kidney and liver (Supplementary Figure S2, S3) demonstrated improvement of diabetes-associated alterations without additional pathological lesions attributable to the nano-formulation. These findings suggest that the ACPO nano-formulation was well tolerated at the administered dose throughout the experimental period.

In the present study, metformin exhibited a more pronounced hypoglycemic effect than the ACPO nano-formulation, as evidenced by the greater reduction in blood glucose levels. However, despite its comparatively modest glucose-lowering effect, the ACPO nano-formulation produced comparable improvements in histopathological architecture and caspase-3 immunoexpression, while demonstrating greater modulation of several molecular markers involved in neuronal survival, oxidative stress, and neuroinflammation. These findings suggest that the neuroprotective effects of the ACPO nano-formulation are not solely dependent on glycemic control but may also result from direct modulation of cellular signaling pathways implicated in diabetes-induced neuronal injury**.** The neuroprotective response observed with the ACPO nano-formulation may therefore reflect the combined effects of its bioactive composition and enhanced formulation characteristics. However, further studies incorporating protein-level validation, pharmacokinetic and biodistribution analyses, and direct assessment of BBB permeability will be valuable for further defining the molecular and cerebral delivery characteristics of the ACPO nano-formulation and its potential clinical applicability. The present study provides evidence for the neuroprotective effects of ACPO nano-formulation against diabetes-associated brain alterations. However, several aspects warrant further investigation. The molecular findings were based on gene-expression analysis, and future studies incorporating protein-level and phosphorylation assessments would provide further insight into the functional status of the PI3K/Akt/mTOR pathway. In addition, pharmacokinetic and brain-biodistribution studies would be valuable for determining the brain availability and distribution of ACPO bioactive constituents following nanoencapsulation. Furthermore, because the interventions were initiated before diabetes induction and continued thereafter, future studies in which treatment is initiated after establishment of diabetes would help further define the therapeutic potential of the nano-formulation. Addressing these aspects in future investigations would further strengthen the mechanistic understanding and translational potential of ACPO nano-formulation in diabetes-associated brain dysfunction.

## Conclusion

The present study demonstrated that the ACPO nano-formulation exerts neuroprotective effects against diabetes-induced brain injury through modulation of multiple molecular pathways involved in neuronal survival, oxidative stress, apoptosis, and neuroinflammation. Compared with metformin, the nano-formulation produced greater modulation of several molecular markers, including higher Akt, mTOR, GPx, and SOD gene expression together with lower GSK3β, PANX1, and AChE gene expression. Although free ACPO oil induced the highest PI3K gene expression, the nano-formulation produced a more pronounced modulation of PI3K/Akt/mTOR pathway-related gene expression. These molecular effects were accompanied by improved cerebral histopathology and reduced caspase-3 immunoexpression, supporting an anti-apoptotic effect of ACPO and its nano-formulation. Overall, these findings suggest that nanoencapsulation enhances the molecular neuroprotective efficacy of ACPO, highlighting its potential as a promising approach for mitigating diabetes-associated brain dysfunction.

## Supplementary Information

Below is the link to the electronic supplementary material.

Supplementary file1 (DOCX 2568 KB)

## Data Availability

The datasets generated and analyzed during the current study are available from the corresponding author upon reasonable request.
