# Supplementary material for: Avocado Oil Nanoemulsion Ameliorates STZ-Induced Diabetic Brain Dysfunction through PI3K/AKT/mTOR-Related Gene Modulation and Attenuation of Apoptosis
Source: Mol Neurobiol. 2026 Nov 4;63(1):1010. doi: 10.1007/s12035-026-06238-4 (PMC13736447; doi:10.1007/s12035-026-06238-4)
Supplement: Supplementary file 1 [file 12035_2026_6238_MOESM1_ESM.docx]

**Supplementary materials**

This supplementary material is provided in response to **Reviewer 3, Comment 3**, regarding the evaluation of organ specificity and off-target toxicity of the ACPO nano-formulation.

Supplementary Figure S1. Serum biochemical parameters related to liver and kidney function

**Supplementary Figure S1. Serum biochemical parameters related to liver and kidney function.** (a) Serum ALT levels, (b) serum AST levels, (c) serum urea levels, and (d) serum creatinine levels. Data are presented as **mean ± SEM** (n = 7 rats per group). Columns with different lowercase letters indicate statistically significant differences (*P* < 0.05). NC: normal control; BC: blank control; RC: reference drug control; OC: oil control; FC: formula control; PC: positive control; BT: blank treated; RT: reference drug-treated; OT: oil-treated; FT: formula-treated.

**Results description:**

(a) **Serum ALT levels were significantly elevated in the diabetic positive control group compared with all treated groups. Both the ACPO nano-formulation and metformin significantly reduced ALT levels to comparable values and were significantly more effective than ACPO oil.**
(b) **Serum AST levels were significantly increased in the diabetic positive control group. Treatment with the ACPO nano-formulation and ACPO oil significantly reduced AST levels to a similar extent, whereas metformin produced a partial reduction that remained significantly higher than both ACPO-treated groups.**
(c) **Serum urea levels were significantly increased in the diabetic positive control group.Treatment with the ACPO nano-formulation and ACPO oil significantly reduced urea levels to a similar extent, whereas metformin produced a partial reduction that remained significantly higher than both ACPO-treated groups.**
(d) **Serum creatinine levels were significantly elevated in the PC group compared with the NC, BC, RC, OC, FC, OT, and FT groups (P < 0.05), whereas no significant difference was observed between the PC, BT, and RT groups. Although serum creatinine levels were numerically lower in the OT and FT groups than in the RT group**

**2. Histopathological evaluation of the kidney**

Histopathological examination of the kidneys demonstrated normal renal architecture in the NC, BC, RC, OC, and FC groups. Severe renal lipidosis involving the tubular epithelial cells was observed in the PC and BT groups. The RT and OT groups showed moderate degeneration of the tubular epithelium, whereas the FT group exhibited only mild renal lipidosis affecting a few tubular epithelial cells. Semiquantitative histopathological scoring revealed a significant reduction in renal lesion severity in all treated diabetic groups compared with the untreated diabetic groups (P < 0.05). The FT group showed lesion scores comparable to those of the normal control group and significantly lower than those of the untreated diabetic groups (Fig. 2k). No evidence of treatment-related renal histopathological lesions was observed in the FT group.

Figure 2. Representative photomicrographs of the renal cortex in the experimental groups. (a) NC, (b) BC, (c) RC, (d) OC, and (e) FC showed normal renal histological structure. (f) PC and (i) BT groups exhibited severe renal lipidosis of the tubular epithelial cells (black arrows). (g) RT and (h) OT groups showed moderate degeneration of the epithelial lining of some renal tubules. (j) FT group showed only mild renal lipidosis affecting a few tubular epithelial cells (black arrows). Hematoxylin and eosin (H&E), ×200. (k) Histopathological scores are expressed as median (IQR). Different lowercase letters indicate statistically significant differences among groups according to the Kruskal–Wallis test followed by Dunn's multiple comparisons test (P < 0.05). Groups sharing at least one letter are not significantly different.

**3. Histopathological evaluation of the liver**

Histopathological examination of the liver revealed normal hepatic architecture in the NC, BC, RC, OC, and FC groups. In contrast, the PC and BT groups exhibited marked hepatic lesions characterized by hepatocellular vacuolar degeneration, Kupffer cell activation, portal mononuclear inflammatory cell infiltration, solitary hepatocellular necrosis, and congestion of the central vein. The RT and OT groups showed marked histological improvement, with only mild residual hepatic alterations. The FT group exhibited nearly normal hepatic architecture with only occasional binucleated hepatocytes and no evidence of treatment-related hepatic lesions. Semiquantitative histopathological scoring demonstrated a significant reduction in hepatic lesion severity in all treated diabetic groups compared with the untreated diabetic groups (P < 0.05). The FT group showed lesion scores comparable to those of the normal control group and significantly lower than those of the untreated diabetic groups (Fig. 3k).

Figure 3. Representative photomicrographs of the liver in the experimental groups. (a) NC, (b) BC, (c) RC, (d) OC, and (e) FC showed normal histological structure. (f) PC and (g) BT groups showed marked mononuclear inflammatory cells in portal area (black arrow), binucleation of some hepatocytes (red arrow), solitary hepatic necrosis (green arrow), Kupffer cell activation (yellow arrow), vacuolar degeneration of some hepatocytes (blue arrow), and congestion of central vein (gray arrow). (h) RT showed binucleation of some hepatocytes, (i) OT groups showed vacuolation of some hepatocytes (blue arrow) and Kupffer cell activation (yellow arrow), (j) FT group showed binucleation of some hepatocytes (red arrow). Hematoxylin and eosin (H&E), ×200. (k) Histopathological scores are expressed as median (IQR). Different lowercase letters indicate statistically significant differences among groups according to the Kruskal–Wallis test followed by Dunn's multiple comparisons test (P < 0.05). Groups sharing at least one letter are not significantly different.
